# Editorial: Novel Concepts in Mechanisms Modulating HBV Persistence, Pathogenesis, and Oncogenetic Properties

**DOI:** 10.3389/fmicb.2021.822813

**Published:** 2021-12-24

**Authors:** Valentina Svicher, Loredana Sarmati

**Affiliations:** ^1^Department of Biology, University of Rome Tor Vergata, Rome, Italy; ^2^Unit of Infectious Disease, University Hospital Tor Vergata, Rome, Italy

**Keywords:** HBV - hepatitis B virus, occult infection, chronic infection, hepatocarcinogenesis, HBsAg

According to recent estimates, 2 billion individuals have been infected by hepatitis B virus (HBV). Among them, 250 million have developed a chronic infection that can lead to end-stage liver diseases, causing around 1 million death per year (World Health Organization, 2021).

After the entry into the hepatocytes, HBV genome migrates into the nucleus where it is converted in the so-called circular covalently closed DNA (cccDNA). cccDNA acts as a mini-chromosome, enabling HBV to establish persistent infections either in the form of chronic or occult infection. In the productive infection, cccDNA is the template for the synthesis of viral mRNAs and in turn of viral proteins whose massive release from infected hepatocytes is recognized as a mechanism contributing to the exhaustion of immune response against the virus, leading to the chronicity of HBV infection. The currently available anti-HBV drugs can efficiently suppress viral replication but cannot directly affect the burden and the transcriptional activity of cccDNA. In this special issue, the fine mechanisms underlying HBV persistence have been comprehensively and clearly described in the review by Gosh et al. As emphasized by the authors, deciphering such mechanisms is critical in order to identify novel therapeutic targets aimed at achieving HBV cure.

HBV persistence is also an important driver of hepatocarcinogenesis. Intriguingly, unlike other etiologies, HBV is endowed by direct pro-oncogenetic properties that can promote the neoplastic transformation of the hepatocytes even in the absence of liver inflammation (Wang et al., 2021). Unfortunately, the currently available anti-HBV drugs can reduce but not abolish the risk to develop hepatocellular carcinoma (HCC) (Tseng et al., 2020; Yip et al., 2020). Another important aspect to consider is that HBV-driven hepatocellular carcinoma (HCC) is frequently diagnosed at an advanced stage, when treatment options are limited and the mortality rate is high. On these bases, the availability of accurate serum biomarkers, that can reflect the extent of intrahepatic HBV reservoir and its oncogenic potential, is critical to identify patients more prone to develop HCC deserving more intense liver monitoring. These biomarkers have been accurately described in the review by Liu et al. carefully analyzing the role of viral proteins (including HBsAg isoforms and HBcrAg), serum HBV-DNA and RNA in predicting not only the occurrence but also the recurrence of HCC. The authors highlight the importance to incorporate selected viral biomarkers into HCC risk algorithms, improving health outcomes for the large amount of people worldwide living with chronic HBV infection.

Interestingly, in this special issue, the review by Coffin et al. has dedicated the attention to HBV lymphotropism, an intriguing aspect of HBV infection that deserves to be deepened. Notably, the review elegantly provides evidence on HBV capability to propagate within immune cells (even during successful antiviral treatment) and to give origin to viral strains with genetic profiles different than those observed in the liver, some of which associated with enhanced risk of HCC development (Lau et al., 2020). The review also addresses the issue of HBV-DNA integration in lymphoid cells that could contribute to the development of some forms of lymphoma (particularly diffuse large B cell lymphoma) observed in patients with chronic HBV infection (Li et al., 2018; Su et al., 2019; Zhou et al., 2019). Finally, HBV-infected lymphoid cells can also contribute to the reinfection of the liver in patients undergoing liver transplantation (Coffin et al., 2015).

Beyond chronic infection, HBV can establish occult infection in which immune responses suppress cccDNA transcriptional activity, favoring the entry into a latent or minimally-replicating status (Raimondo et al., 2019). Occult HBV infection is intensively investigated since it can give origin to HBV reactivation during conditions of immunesuppression and can act as an important cofactor in the onset of hepatocellular carcinoma (Raimondo et al., 2019). Understanding mechanisms underlying the establishment of OBI is still a matter of debate. In this light, the study by Wang et al. has investigated the role of genetic variability in the N-terminus of HBsAg in mechanisms underlying HBsAg-negativity, a typical feature of OBI. The authors found that mutations at the HBsAg position 2 can hamper HBsAg secretion, presumably by impeding the proper HBsAg folding in the membrane of the endoplasmic reticulum, thus contributing to explain HBsAg-negativity observed in patients with OBI. Notably, this result is stronger in genotype C than B, suggesting that the genetic backbone of HBV genotypes can modulate mechanisms underlying the establishment of OBI. In the same direction of research, the study by Ou et al. has focused the attention on the role of large surface glycoprotein in HBV infectivity and morphogenesis. The authors focused on the pre-S1 domain (located at the N-terminus), highlighting its important role in modulating, in a genotype-specific manner, the infectivity and replicative capacity of mature virions as well as the secretion of subviral particles. Again, this reinforces the different HBV replicative and pathogenetic potential of HBV genotypes, highlighting the need to consider the influence of HBV genotypes in antiviral drug screening, especially for entry inhibitors.

## Author Contributions

Both authors listed have made a substantial, direct, and intellectual contribution to the work and approved it for publication.

## Conflict of Interest

The authors declare that the research was conducted in the absence of any commercial or financial relationships that could be construed as a potential conflict of interest.

## Publisher's Note

All claims expressed in this article are solely those of the authors and do not necessarily represent those of their affiliated organizations, or those of the publisher, the editors and the reviewers. Any product that may be evaluated in this article, or claim that may be made by its manufacturer, is not guaranteed or endorsed by the publisher.
